# *De Novo* Mutation in Non-Tyrosine Kinase Domain of *ROS1* as a Potential Predictor of Immune Checkpoint Inhibitors in Melanoma

**DOI:** 10.3389/fonc.2021.666145

**Published:** 2021-06-17

**Authors:** Si-Cong Ma, Hong-Bo Zhu, Jian Wang, Yan-Pei Zhang, Xue-Jun Guo, Li-Li Long, Ze-Qin Guo, De-Hua Wu, Zhong-Yi Dong, Xue Bai

**Affiliations:** ^1^ Department of Radiation Oncology, Nanfang Hospital, Southern Medical University, Guangzhou, China; ^2^ Information Management and Big Data Center, Nanfang Hospital, Southern Medical University, Guangzhou, China; ^3^ Department of Oncology, The First Affiliated Hospital of University of South China, Hengyang, China

**Keywords:** *ROS1* mutation, immune checkpoint inhibitor, melanoma, tyrosine kinase domain, tumor mutational burden

## Abstract

**Purpose:**

Despite the success of targeted therapy in c-ros oncogene 1 (*ROS1*)-rearranged cancers, especially non-small cell lung cancer (NSCLC), the clinical significance of *ROS1 de novo* mutation has not yet been understood. We sought to elucidate the predictive effect of *ROS1* mutation for immune checkpoint inhibitor (ICI) therapy in melanoma.

**Methods:**

The Cancer Genome Atlas [TCGA (*n* = 10967)] and Memorial Sloan Kettering Cancer Center [MSK (*n* = 10,945)] datasets, as well as two clinical cohorts of melanoma received ICI [CA209-038 (*n* = 73) and MEL-IPI (*n* = 110)], were included to explore the prevalence, prognostic effect, and immunotherapeutic predictive effect of *ROS1* mutation in melanoma. Overall survival (OS) was defined as the primary outcome.

**Results:**

Overall, melanoma accounted for the highest proportion of *ROS1* mutation (~20%) which made up the majority (~95%) of the *ROS1*-alterated cases. Remarkably, *ROS1* mutation yielded longer OS from ICI than the wild-type counterpart in the MSK melanoma population [hazard ratio (HR) 0.47, 95% confidence interval (CI) 0.30–0.74], and two external melanoma cohorts (CA209-038: HR 0.42, 95% CI 0.20–0.89; MEL-IPI: HR 0.55, 95% CI 0.34–0.91), without affecting the prognosis of patients. Elevated tumor mutational burden and enrichment of DNA damage repair was observed in *ROS1* mutated patients, providing an explanation for the favorable responses to ICI therapy. Precisely, *ROS1* mutation in non-protein tyrosine kinase (PTK) domain but not PTK mutation was responsible for the immunotherapy-specific responses of the *ROS1* mutated patients in melanoma.

**Conclusions:**

Collectively, *ROS1* mutation, specifically the non-PTK mutation, is a potential predictor of ICI therapy in melanoma, which is distinct from the well-established role of *ROS1* rearrangement for targeted therapy in NSCLC.

## Highlights

- Melanoma accounted for the highest proportion of *ROS1* mutation which made up the majority of the *ROS1*-alterated cases.
- *ROS1* mutation served as a favorable predictor for immune checkpoint therapy but not a prognostic factor in melanoma.
- *ROS1* mutation was correlated with elevated tumor antigenicity and genomic instability.
- The non-PTK domain was the specific site of *ROS1* mutation that determine the favorable responses to immune checkpoint therapy in melanoma.

## Introduction

The c-ros oncogene 1 (*ROS1*) gene has aroused great research interest for its role as an oncogenic driver of malignancies, as well as its untapped potential for novel therapeutic targets (1). It encodes a receptor tyrosine kinase with a tyrosine kinase domain located in the intracellular C-terminal (2). Long-term efforts have been devoted to develop tyrosine kinase inhibitors targeting the continuously activated *ROS1* proteins resulted by chromosomal rearrangements, and crizotinib has received approval of the U.S. Food and Drug Administration (FDA) for *ROS1*-rearranged cancers (3). Nonetheless, almost all previous studies focused specifically on *ROS1*-rearranged non-small cell lung cancer (NSCLC), which only makes up a tiny percentage of patients (4). The focus on *ROS1*-rearranged NSCLC on the one hand contributes to the tremendous success of tyrosine kinase inhibitors targeting the *ROS1* rearrangement, whereas on the other hand leads to the neglect of the therapeutic potential behind the *ROS1* alterations other than rearrangement, particularly *de novo* mutation that accounts for a larger proportion.

With the unprecedented progress of immune checkpoint inhibitors (ICIs), immunotherapy has altered the treatment paradigm and gradually become the mainstay of the treatment for advanced cancers, especially melanoma (5, 6). Identifying marker of significance to predict responses to ICI therapy has become a key challenge in study and clinical practice of immunotherapy because of the heterogeneity of ICI effectiveness seen among patients in the clinical work (7–11). Given the relatively modest predictive power of the routinely applied immunotherapeutic biomarkers, such as PD-L1, growing studies have been committed to search for clues from gene mutation (8, 12–14). Recent advances have expanded the role of oncogenic driver genes from targeted therapy to immunotherapy, including *STK11*, *EGFR* and *KRAS* (14–16); however, for our knowledge, there were few studies on variations other than rearrangement concerning *ROS1*, and what role the *ROS1* mutation would act in ICI treatment of melanoma has not yet been elucidated to date. Therefore, the purpose of our study was to explore the predictive value of *ROS1* mutation, instead of rearrangement, for the efficacy of ICI treatment in melanoma.

## Methods

### Study Populations

Clinical and genomic data of pan-cancer patients from The Cancer Genome Atlas (TCGA, *n* = 10,967) and Memorial Sloan Kettering Cancer Center (MSK, *n* = 10,945) were retrieved from cBioPortal (http://www.cbioportal.org/). Among the MSK dataset, 1,661 patients were treated with anti-programmed cell death-1 (PD-1) alone or in combination with anti-Cytotoxic Lymphocyte Antigen-4 (CTLA-4). Additionally, two clinical cohorts of melanoma received anti-PD-1 (CA209-038, *n* = 73) or anti-CTLA-4 therapy (MEL-IPI, *n* = 110), along with their corresponding DNA sequencing data, were collected from previously published studies (17, 18). This study was approved by the Institutional Ethical Review Boards of Nanfang Hospital. Patients included in the clinical cohorts have provided signed informed consent in accordance with their corresponding clinical study protocols.

### Study Design

A flow chart showing the study design was demonstrated in **Supplementary Figure 1**. In this study, we first conducted a pan-cancer analysis in the TCGA and the MSK datasets to explore the prevalence of *ROS1* mutation in various cancers. Then, an exploratory analysis was performed to investigate the predictive value of *ROS1* mutation in the MSK ICI-treated population, as well as the prognostic value in the non-ICI treated population. The predictive significance of *ROS1* mutation for ICI therapy in melanoma was furtherly validated in the CA209-038 and the MEL-IPI cohorts. The correlation of *ROS1* mutation status with tumor mutational burden (TMB) was analyzed, and pathway enrichment analysis according to *ROS1* mutation status was conducted to elucidate the potential mechanism. Moreover, immunotherapy-specific survival outcomes were compared between patients harboring *ROS1* mutation in or out of the tyrosine kinase domain to identify the exact mutation sites that predict efficacy of ICI therapy. Overall survival (OS) was defined as the primary outcome in this study.

### Genomic Analysis

Patients in the TCGA dataset, as well as the CA209-038 and the MEL-IPI cohorts were molecularly profiled with whole exome sequencing (WES), while those in the MSK dataset underwent Memorial Sloan Kettering-Integrated Mutation Profiling of Actionable Cancer Targets (MSK-IMPACT), a targeted next-generation sequencing assay (19). TMB was calculated as the absolute count of non-synonymous mutations (including nonsense, missense, nonstop, frame shift, in frame, splice site, and translation start site mutations) by WES, or mutations per megabase by MSK-IMPACT. Tyrosine kinase domain of *ROS1* was identified and visualized within a lollipop chart using cBioportal (20).

### Pathway Enrichment and Network Analysis With RNA Sequencing Data

RNA sequencing data of TCGA melanoma dataset obtained from cBioportal was preprocessed and normalized using RNA-Seq by Expectation Maximization (RSEM) (20, 21). Patients were classified into subgroups according to *ROS1* mutation status. Gene Set Enrichment Analysis (GSEA) was conducted with the java-based GSEA v4.0.3 application to compare the activity of biological processes or pathways between *ROS1* mutation and wild-type (22), using the C2 curated gene sets downloaded from the Molecular Signatures Database (MSigDB) (23). Nominal *P* value evaluated the statistical significance of the enrichment score of a certain pathway or biological process, while false discovery rate (FDR), which was adjusted for gene set size, was estimated to represent the false positive probability. Normalized enrichment score (NES) was used to compare analysis results across gene sets (22). Molecular interaction network was built using STRING (version 11.0), with a minimum required interaction score of 0.99 (24).

### Statistical Analysis

GraphPad Prism (version 8.0.1) or R (version 3.6.1) were used for statistical analyses. Survival analyses were performed using the Kaplan–Meier method with log-rank test. Kaplan–Meier survival curves were plotted using GraphPad Prism or the R packages *survival* and *surminer*; hazard ratios (HRs) with 95% confidence intervals (CIs) were reported. Differential analysis of *ROS1* mutation versus wild-type was conducted using the R package *limma*. TMB and gene expression were compared between subgroups using unpaired t-test. All *P* values were two-sided, and *P* ≤0.05 was considered statistically significant.

## Results

### Frequent *ROS1* Mutation in Melanoma

**Figure 1:**
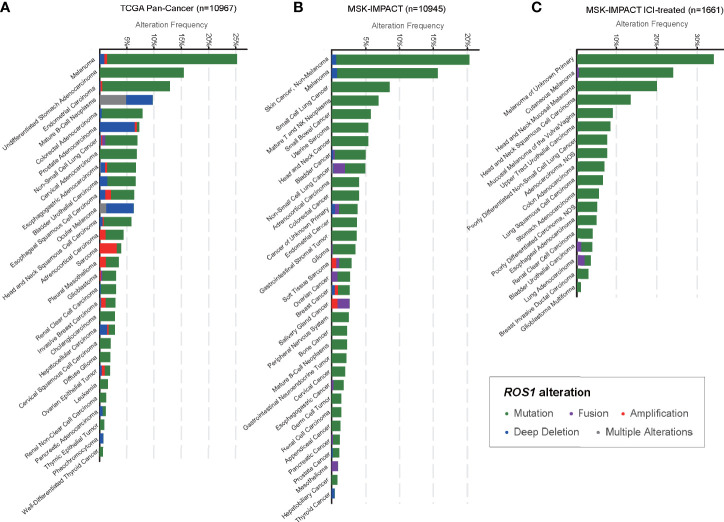
Prevalence of *ROS1* mutation across various cancers. Frequencies of *ROS1* alterations (including mutation, fusion, amplification, deep deletion, and multiple alterations) in various cancer types in **(A)** the TCGA and **(B)** the MSK datasets, and **(C)** detailed cancer subtypes in the MSK ICI-treated population were illustrated. TCGA, The Cancer Genome Atlas; MSK, Memorial Sloan Kettering; ICI, immune checkpoint inhibitor.

In total, we included 10,967 patients from TCGA and 10,945 patients from MSK, among which 1,661 patients were treated with ICI, for genomic analysis evaluating the prevalence of *ROS1* mutation across various cancers (**Figure 1**). Overall, patients with melanoma harbored a predominant prevalence of *ROS1* mutation, with proportions of 25.0 and 14.8% respectively in the TCGA and the MSK datasets (**Figures 1A, B**). Specifically, for the ICI-treated patients of the MSK dataset, *ROS1* mutation was detected in 20.0% of melanoma patients, and the high mutation frequency was also confirmed within different subtypes of melanoma, including cutaneous melanoma, head and neck mucosal melanoma, and melanoma of unknown primary (**Figure 1C**). Notably, *ROS1* mutation accounted for the majority of the *ROS1*-alterated melanoma cases in both datasets (TCGA: 94.6%; MSK: 94.7%). In contrast, *ROS1* fusion only accounted for a small fraction of patients in NSCLC (<2% in both datasets), and even rare in other tumor types (**Figures 1A, B**).

### *ROS1* Mutation Predicts Efficacy of Immunotherapy in Melanoma

**Figure 2:**
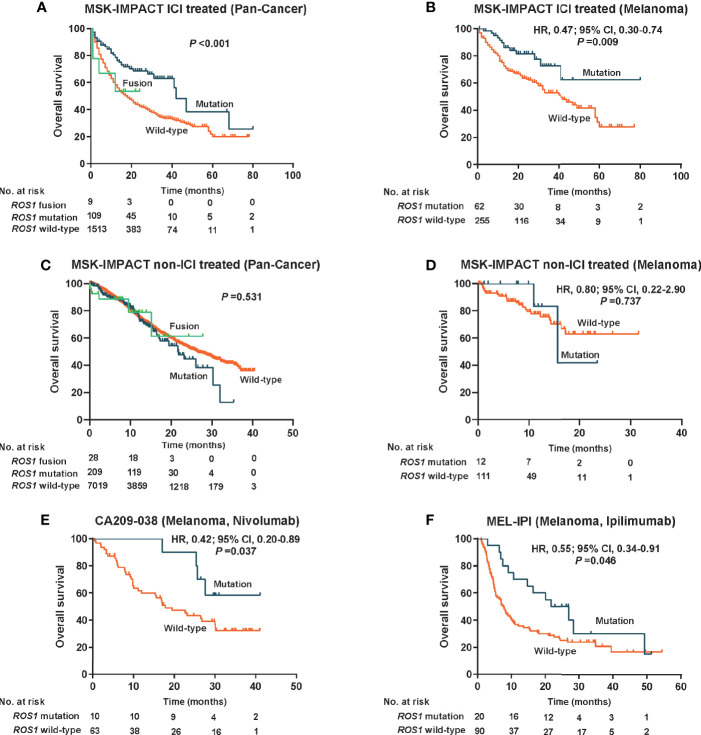
Predictive value for immune checkpoint inhibitors therapy and prognostic effect of *ROS1* mutation. Kaplan–Meier curves of overall survival in **(A)** the pooled MSK ICI-treated population, **(B)** the MSK ICI-treated melanoma population, **(C)** the pooled MSK non-ICI treated population, **(D)** the MSK non-ICI treated melanoma population, **(E)** the CA209-038 cohort of melanoma received nivolumab, and **(F)** the MEL-IPI cohort of melanoma received ipilimumab. MSK, Memorial Sloan Kettering; HR, hazard ratio; CI, confidence interval; ICI, immune checkpoint inhibitor.

Given the high prevalence of *ROS1* mutation in melanoma, we first explored its predictive value for risk stratification of melanoma patients received ICI treatment in the MSK dataset. Remarkably, in the total MSK ICI-treated population, patients harboring *ROS1* mutation obtained longer OS compared to both the fusion subgroup and the wild-type subgroup (*P <*0.001, **Figure 2A**). Specifically, for the ICI-treated melanoma population, survival prospects consistently favored *ROS1* mutation than its wild-type counterpart (HR 0.47, 95% CI 0.30–0.74; *P* = 0.009, **Figure 2B**).

To clarify whether the predictive effect of *ROS1* mutation on immunotherapy was affected by the prognostic effect itself, we evaluated the impact of *ROS1* mutation on prognosis in the non-ICI treated population from the MSK dataset. Generally, no significant difference in OS was observed among the *ROS1* mutation, the wild-type, and the fusion subgroups in the pooled population (*P* = 0.531, **Figure 2C**); likewise, as for the non-ICI treated melanoma patients, *ROS1* mutation demonstrated similar OS as the wild-type counterpart (HR 0.80, 95% CI 0.22–2.90; *P* = 0.737, **Figure 2D**). A consistent finding seen in the TCGA melanoma population (HR 1.15, 95% CI 0.80–1.65; *P* = 0.463, **Supplementary Figure 2**) further confirmed that *ROS1* mutation serves as a potential predictive biomarker of ICI treatment for melanoma, without affecting the prognosis of patients.

Moreover, external validation of the predictive significance of *ROS1* mutation was conducted in two independent ICI-treated melanoma cohorts. Of note, the presence of *ROS1* mutation was associated with long-term survival prospects and increased likelihood of responses to nivolumab in terms of OS in the CA209-038 cohort (HR 0.42, 95% CI 0.20–0.89; *P* = 0.037, **Figure 2E**). The pronounced clinical benefits derived from *ROS1* mutation versus wild type were furtherly confirmed in the MEL-IPI cohort of melanoma treated with ipilimumab (HR 0.55, 95% CI 0.34–0.91; *P* = 0.046, **Figure 2F**).

### *ROS1* Mutation Correlates With Upregulated Tumor Antigenicity and DNA Damage Repair

**Figure 3:**
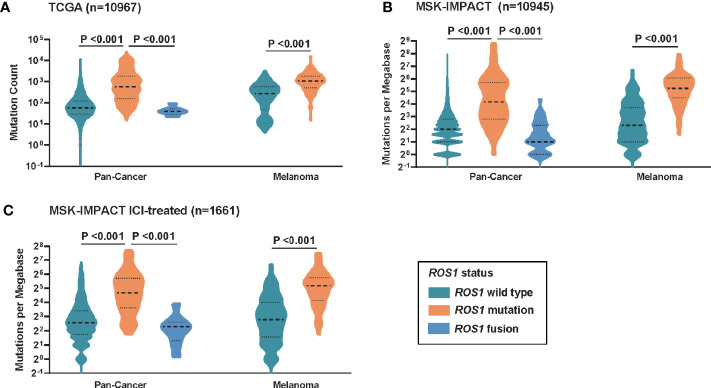
Correlation between *ROS1* mutation status and tumor mutational burden. Distribution of TMB in the total population and the melanoma population in **(A)** the TCGA dataset, **(B)** the MSK dataset, and **(C)** the MSK ICI-treated population. TCGA, The Cancer Genome Atlas; MSK, Memorial Sloan Kettering; ICI, immune checkpoint inhibitor; TMB, tumor mutational burden.

To investigate the potential mechanism behind the predictive power of *ROS1* mutation for ICI therapy, correlation analysis between *ROS1* mutation status and TMB was performed. For the total population, patients with *ROS1* mutation harbored significantly higher TMB than *ROS1* fusion and wild-type patients in both the TCGA and MSK cohorts (all *P <*0.001, **Figures 3A, B**). In parallel, *ROS1* mutation was associated with higher TMB relative to the wild-type counterpart in the melanoma population (all *P <*0.001, **Figures 3A, B**). Equivalent findings were also seen in the MSK ICI-treated patients (all *P <*0.001, **Figure 3C**), providing an explanation for the favorable responses of *ROS1*-mutated patients to ICI therapy.

**Figure 4:**
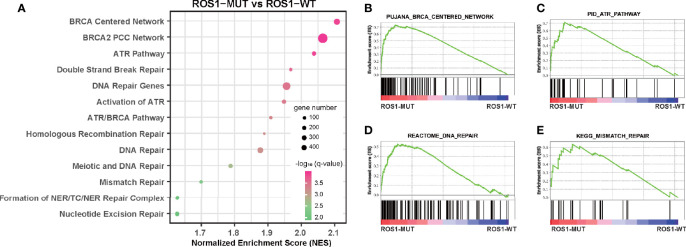
Gene set enrichment analysis of *ROS1* mutation versus wild-type. **(A)** Bubble plot showing the enrichment of DNA damage repair-related pathways in *ROS1* mutation patients relative to wild-type patients in the TCGA melanoma dataset. Enrichment plots of *ROS1* mutation versus wild-type regarding **(B)** BRCA centered network, **(C)** ATR pathway, **(D)** DNA repair, and **(E)** mismatch repair. FDR, false discovery rate; MUT, mutation; WT, wild-type; TCGA, The Cancer Genome Atlas.

Furthermore, we performed GSEA to explain, from the perspective of transcriptomics, the immunotherapy-specific responses of *ROS1* mutation versus wild-type using the RNA sequencing data of the TCGA melanoma dataset (**Figure 4A** and **Supplementary Table 1**). We observed significant enrichment of DNA damage repair (DDR)-related pathways in the subgroup with *ROS1* mutation compared to the wild-type counterpart, including BRCA-centered network, ATP pathway, DNA repair pathway, and mismatch repair pathway (**Figures 4B–E**), indicative of an upregulation of the activity of DDR-related pathways secondary to the accumulation of gene damage and the elevated tumor antigenicity in the *ROS1* mutated population. As a contrast, expression levels of immune checkpoint genes (e.g., *PDCD1*, *CD274*, and *CTLA4*) were not associated with *ROS1* mutation status (**Supplementary Figure 3**). These finding suggest that favorable responsiveness of *ROS1* mutation to ICI therapy was specifically attributed to the elevated tumor antigenicity instead of the expression changes in immune checkpoint molecules.

### Non-Tyrosine Kinase Mutation of *ROS1* as the Precise Subtype of Immunotherapy-Specific Responses

**Figure 5:**
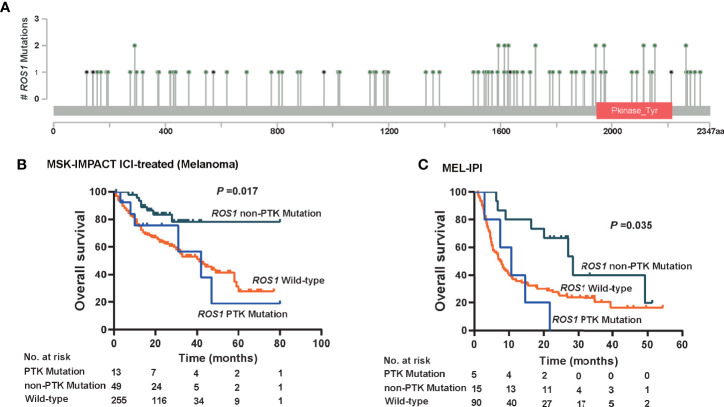
Immunotherapeutic predictive effect of *ROS1* mutation in or out of the protein tyrosine kinase domain. **(A)** Lollipop chart showing the location of the protein tyrosine kinase (PTK) domain of the *ROS1* gene, and mutation number of each position in the MSK ICI-treated melanoma dataset. Kaplan–Meier curves of overall survival in **(B)** the MSK ICI-treated melanoma population, and **(C)** the MEL-IPI cohort of melanoma received ipilimumab. MSK, Memorial Sloan Kettering; ICI, immune checkpoint inhibitor.

Given that patients harboring *ROS1* fusion, which involves the protein tyrosine kinase (PTK) domain, responded unfavorably to ICI therapy (**Figure 2A**), we wonder whether *de novo* mutation of *ROS1* within the PTK domain might also compromise the immunotherapy-specific responses. Therefore, we divided *ROS1* mutated patients into subgroups according to mutation location. Mutations within the PTK domain (amino acid sites from 1,947 to 2,215) were assigned as PTK mutations, whereas those not in this scope were assigned as non-PTK mutation (**Figure 5A**).

Approximately, non-PTK mutation accounted for ~75% of *ROS1* mutation in melanoma patients, namely 79.0% (*n* = 49) in the MSK ICI-treated melanoma population and 75.0% (*n* = 15) in the MEL-IPI cohort (**Figures 5B, C**). It is noteworthy that non-PTK mutation of *ROS1* yielded significantly prolonged OS relative to both *ROS1* PTK mutation and wild type in the MSK ICI-treated melanoma population (*P* = 0.017, **Figure 5B**). In a similar vein, OS was longer in the *ROS1* non-PTK mutation arm relative to the *ROS1* PTK mutation arm and the ROS1 wild-type arm in the MEL-IPI cohort (*P* = 0.035, **Figure 5C**). Taken together, these results indicate that non-PTK mutation of *ROS1* is the precise mutation subtype that predicts efficacy of ICI therapy.

## Discussion

In this genetic association study, we demonstrated a relatively high mutation frequency of *ROS1* in pan-cancer especially melanoma. Notably, *ROS1* mutation served as a favorable predictor for ICI therapy but not a prognostic factor in melanoma, whereas *ROS1* fusion and wild-type patients derived limited benefits from ICI therapy, potentially attributed to the relatively elevated tumor antigenicity and genomic instability in *ROS1* mutated patients. Furthermore, we identified the non-PTK domain as the specific sites of *ROS1* mutation that determine the favorable responses to ICI therapy in melanoma.

As a well-known driver gene, *ROS1* has long been studied; however, to the best of our knowledge, almost all the studies concerning on *ROS1* alterations revolved around chromosomal rearrangement (1, 3, 4). Admittedly, ROS1 targeted inhibitors, represented by crizotinib, achieved tremendous success in *ROS1*-fusion cancers especially NSCLC (3, 4), but actually, rearrangement just accounts for a fairly small proportion of patients, less than 2% in NSCLC, and even lower in other malignancies (1, 25). Therefore, the effective population of the ROS1 inhibitors was quite limited. Even worse, the resistance to ROS1 inhibitors, owing to the mutation of the kinase domain or activation of bypass pathways, makes the circumstances more pickle (26). In these regards, broader investigation probing into the function of *ROS1* alterations, other than rearrangement, is warranted to devise new therapeutic strategies for cancers.

Herein, we identified *ROS1* mutation as a predictor for ICI therapy, enabling people to re-examine the value of the gene as another therapeutic target. Compared with *ROS1* rearrangement in targeted therapy, *ROS1* mutation plays a positive role in ICI therapy. More importantly, it accounts for much greater number of patients than rearrangement, which means more beneficiaries could be identified for precision therapy. Interestingly, *ROS1* fusion and wild type were both found to yield a relatively poor response to ICI therapy, potentially attributed to the low mutational burden which has been shown by previous studies to associate with low neoantigenicity, thus hindering the activity of immune system against tumors (27); hence it was reasonable to recommend those patients with *ROS1* mutation to receive ICI therapy, whereas those with *ROS1* fusion still to receive targeted therapy. Consequently, our finding of *ROS1* mutation in ICI therapy was a strong complement for the treatment of patients with *ROS1* alterations.

Elucidation of the molecular mechanisms of genomics instability and DNA damage repair have pioneered a new avenue for cancer treatment (28); recent studies on the association between gene damage and tumor neoantigenicity have laid the DNA repair processes and DNA damage checkpoints of an important position in ICI-based immunotherapy (27, 29). Our study suggested an enrichment of DDR-related processes and damage sensor protein-centered pathways in the *ROS1* mutated population, represented by upregulation of gene expression of *BRCA1/2*, *ATR*, and *ATM* (**Supplementary Figure 4**). Network interaction analysis of *ROS1* mutation versus wild-type also revealed an enrichment of DNA repair-related molecules (**Supplementary Figure 5**). On this ground, it is envisioned that patients harboring *ROS1* mutation are intrinsically characterized with a hyper mutation status with accumulated genomics instability and gene damage that increased tumor mutational burden, which in turn triggers the secondary activation of DDR-related pathways as well as the resultant immune surveillance at the meantime. In contrast, there was no significant enrichment of biological processes concerning epithelial-mesenchymal transition for *ROS1* mutated subgroup versus the wild-type counterpart, indicative of the irrelevance between *ROS1* mutation and metastasis (**Supplementary Figure 6**).

As *ROS1* mutations are quite heterogeneous, whether mutations in distinct gene loci confer differential responses to ICI therapy is of significance to explore. To this end, we have classified them based on the mutation location, namely mutations in PTK domain and those in non-PTK domain, so as to identify the exact mutation sites of immunotherapy-specific responses. Interestingly, our data illustrated that it was those patients harboring non-PTK mutation instead of PTK mutation that derived significant efficacy benefits from ICI therapy than the wild-type counterparts. With the aforementioned finding, we proposed that the use of ICI might be an ideal treatment option for patients harboring *ROS1* mutation in the non-PTK domain.

This study does have several limitations. First of all, the specific molecular mechanism behind the predictive significance of non-PTK mutation of *ROS1* have not been fully characterized, so more comprehensive biological mechanisms are still to be elucidated in future studies. In addition, as a multicohort study, heterogeneity among cohorts is inevitable; still, despite this objective fact, our results demonstrate consistent tendency across cohorts, which in a sense reflects the credibility of the conclusions. Lastly, due to the retrospective nature of the *post hoc* analysis, it is still necessary to verify the predictive value of *ROS1* mutation, specifically the non-PTK mutation, for ICI therapy in prospective clinical trials.

## Conclusions

In conclusion, we for the first time reveal the positive predictive value of *ROS1* mutation, characterized with increased tumor antigenicity and gene damage, for ICI therapy in melanoma, which is distinct from the well-established role of *ROS1* rearrangement for targeted therapy in NSCLC. Moreover, non-PTK mutation is identified as the precise mutation subtype of immunotherapy-specific responses. Further elucidation of the biological mechanisms and validation in future prospective studies are needed before its application in the clinical practice.

## Data Availability Statement

Publicly available datasets were analyzed in this study. This data can be found here: http://www.cbioportal.org/.

## Ethics Statement

The study was approved by the Nanfang Hospital, Southern Medical University. The patients/participants included in the clinical cohorts have provided signed informed consent in accordance with their corresponding clinical study protocols.

## Author Contributions

Study design: XB, Z-YD, D-HW. Data collection: S-CM, XB, JW, and X-JG. Data analysis and interpretation: Z-YD, S-CM, XB, H-BZ, JW, Y-PZ, L-LG, and Z-QG. Writing of the manuscript: S-CM, XB, and Y-PZ. Revision of the manuscript: Z-YD, D-HW, XB, H-BZ, JW, X-JG, L-LG, and Z-QG. Statistical analysis: Z-YD, S-CM, and H-BZ. All authors contributed to the article and approved the submitted version.

## Funding

This study was supported by the National Natural Science Foundation for Young Scientists of China (Grant Nos. 81802863 and 81902353), the Natural Science Foundation of Guangdong Province (Grant No. 2018030310285), the Outstanding Youths Development Scheme of Nanfang Hospital, Southern Medical University (Grant No. 2017J003 and 2020J011), and the College Students’ Innovative Entrepreneurial Training Plan Program (Grant Nos. 202012121001 and S202012121065).

## Conflict of Interest

The authors declare that the research was conducted in the absence of any commercial or financial relationships that could be construed as a potential conflict of interest.

## Supplementary Material

Supplementary Figure 1 Flow chart of the study design. ICI, immune checkpoint inhibitor; TMB, tumor mutational burden; MSK, Memorial Sloan Kettering; TCGA, The Cancer Genome Atlas.

Supplementary Figure 2 Kaplan Meier curves according to overall survival of *ROS1* MUT *versus* WT in non-ICI treated melanoma patients from the TCGA melanoma dataset. MUT, mutation; WT, wild-type; ICI, immune checkpoint inhibitor; TCGA, The Cancer Genome Atlas.

Supplementary Figure 3 Gene expression levels of **(A)** inhibitory receptors within the PD-1 pathway, and **(B)** other immune checkpoint molecules in The Cancer Genome Atlas (TCGA) melanoma dataset according to the mutation status of *ROS1*.

Supplementary Figure 4 Gene expression levels of representative genes within DNA damage repair-related and damage sensor protein-centered pathways in The Cancer Genome Atlas (TCGA) melanoma dataset according to the mutation status of *ROS1*.

Supplementary Figure 5 Network analysis for *ROS1* downstream mechanisms in TCGA melanoma dataset. **(A)** Volcano plot showing differential analysis of *ROS1* mutation versus wild-type; those genes with P<0.01 were retained for network establishment. **(B)** Interaction network with a minimum required interaction score of 0.99. **(C)** BRCA-centered subnetwork. DNA repair-related molecules were color coded within the network. MUT, mutation; WT, wild-type; TCGA, The Cancer Genome Atlas.

Supplementary Figure 6 Enrichment plots of *ROS1* mutation versus wild-type in biological processes concerning metastasis or epithelial-mesenchymal transition (EMT). NES, normalized enrichment score; FDR, false discovery rate.

## Abbreviations

ICI, immune checkpoint inhibitor; TCGA, The Cancer Genome Atlas; MSK, Memorial Sloan Kettering; PD-1, programmed cell death-1; CTLA-4, Cytotoxic Lymphocyte Antigen-4; TMB, tumor mutational burden; DDR, DNA damage repair; PTK, protein tyrosine kinase; HR, hazard ratio; CI, confidence interval, NSCLC, non-small cell lung cancer; FDA, Food and Drug Administration.
