# Supplementary figures and images for: De Novo Mutation in Non-Tyrosine Kinase Domain of ROS1 as a Potential Predictor of Immune Checkpoint Inhibitors in Melanoma
Source: Front Oncol. 2021 Jun 17;11:666145. doi: 10.3389/fonc.2021.666145 (PMC8247586; doi:10.3389/fonc.2021.666145)

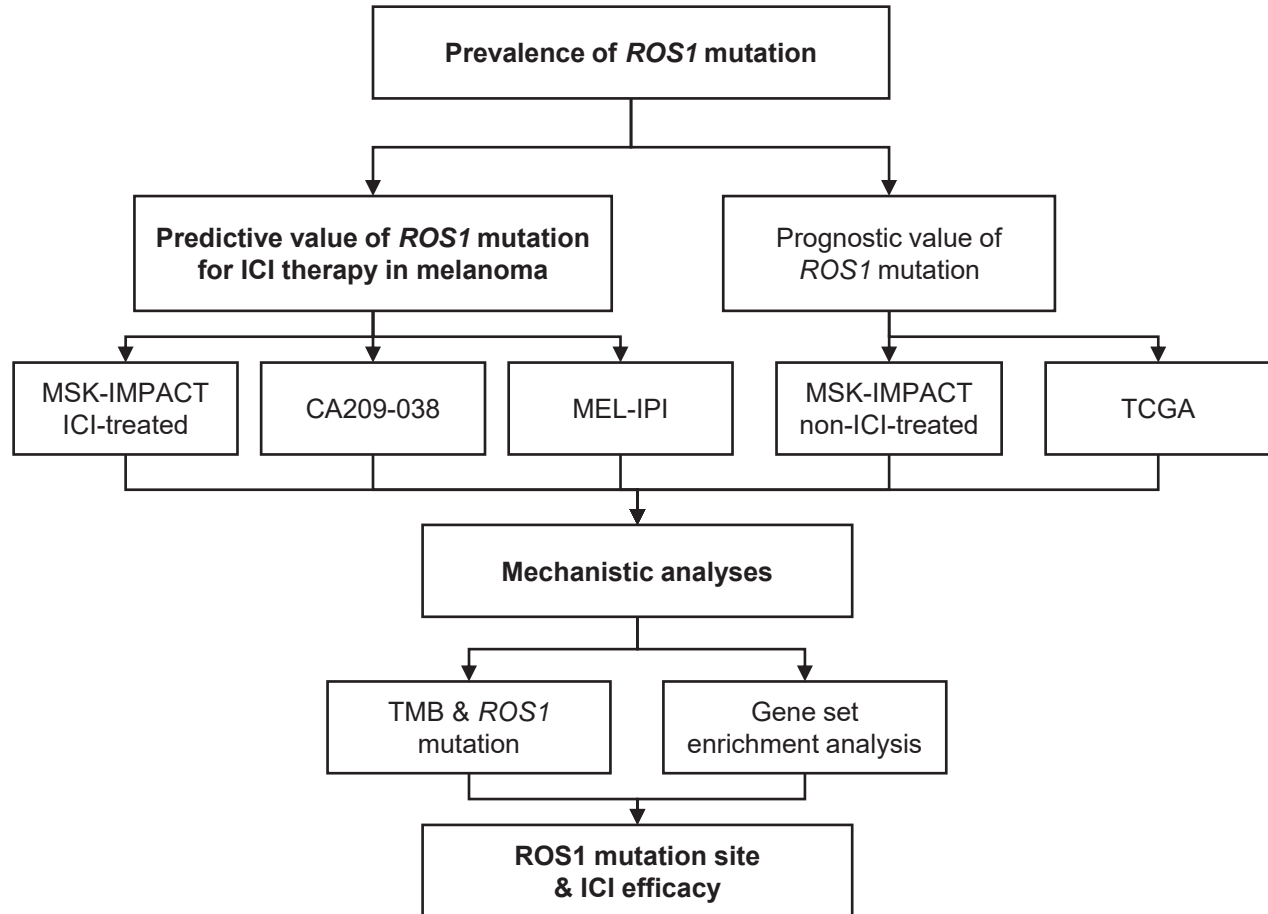

Supplement: Supplementary Figure 1 — Flow chart of the study design. ICI, immune checkpoint inhibitor; TMB, tumor mutational burden; MSK, Memorial Sloan Kettering; TCGA, The Cancer Genome Atlas. [file DataSheet_1.pdf]

Overall Survival

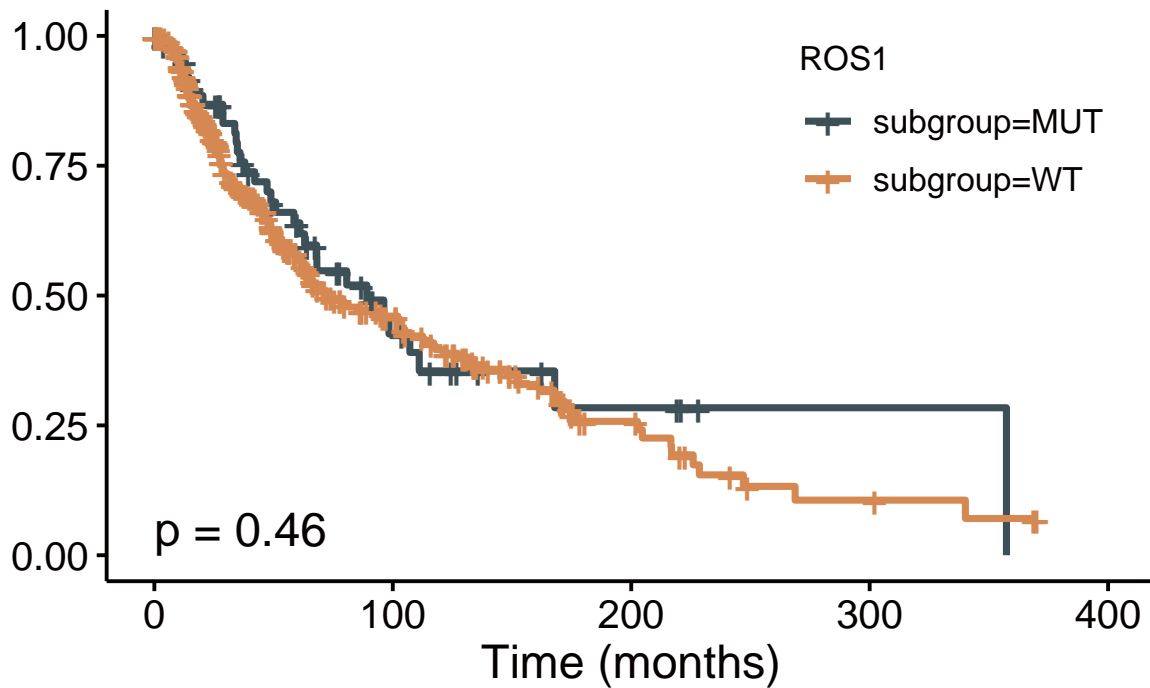

Number at risk

|              |     |    |    |   |   |
|--------------|-----|----|----|---|---|
| subgroup=MUT | 65  | 13 | 4  | 1 | 0 |
| subgroup=WT  | 358 | 75 | 17 | 4 | 0 |

Supplement: Supplementary Figure 2 — Kaplan Meier curves according to overall survival of ROS1 MUT versus WT in non-ICI treated melanoma patients from the TCGA melanoma dataset. MUT, mutation; WT, wild-type; ICI, immune checkpoint inhibitor; TCGA, The Cancer Genome Atlas. [file DataSheet_2.pdf]

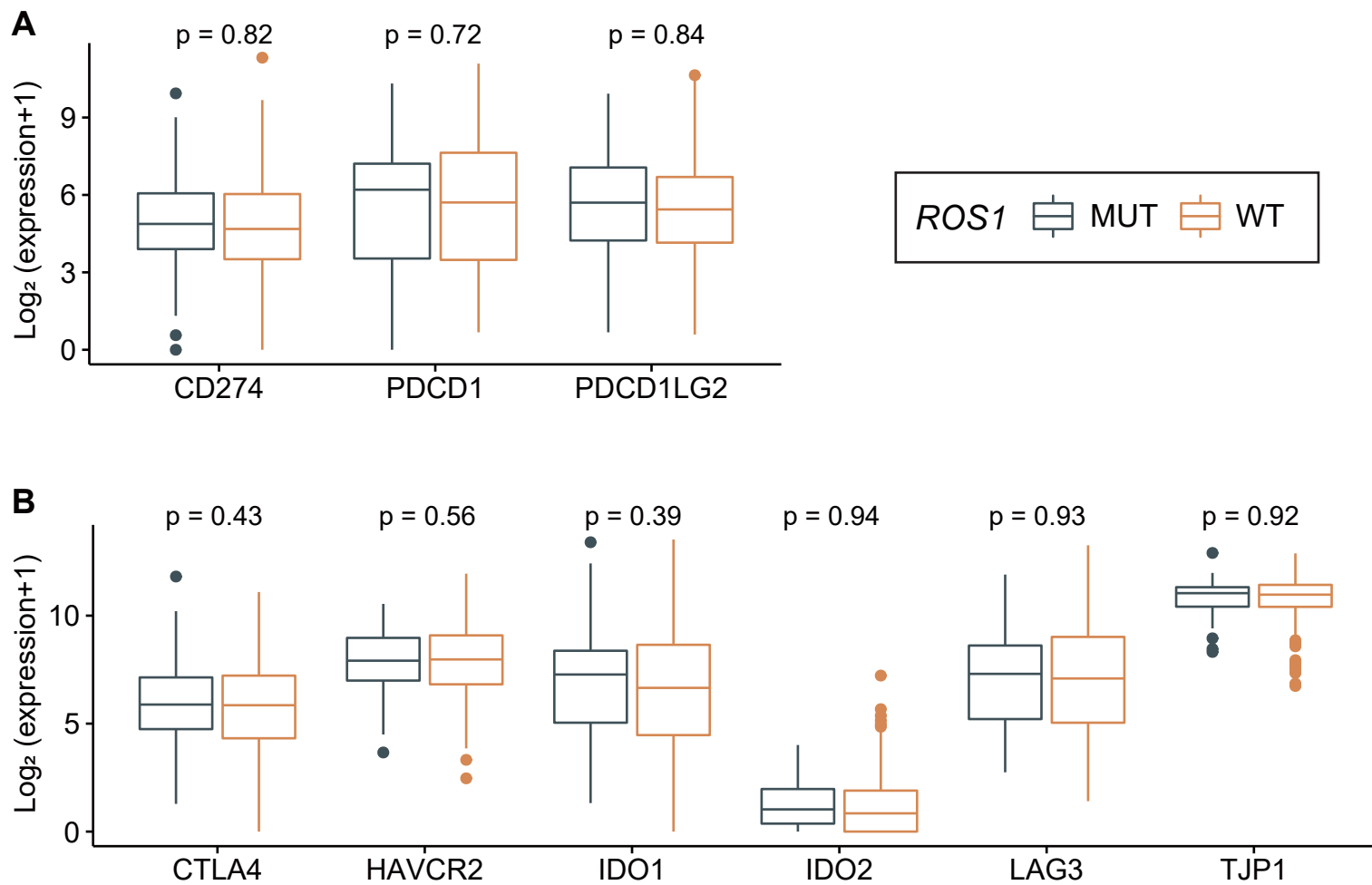

Supplement: Supplementary Figure 3 — Gene expression levels of (A) inhibitory receptors within the PD-1 pathway, and (B) other immune checkpoint molecules in The Cancer Genome Atlas (TCGA) melanoma dataset according to the mutation status of ROS1. [file DataSheet_3.pdf]

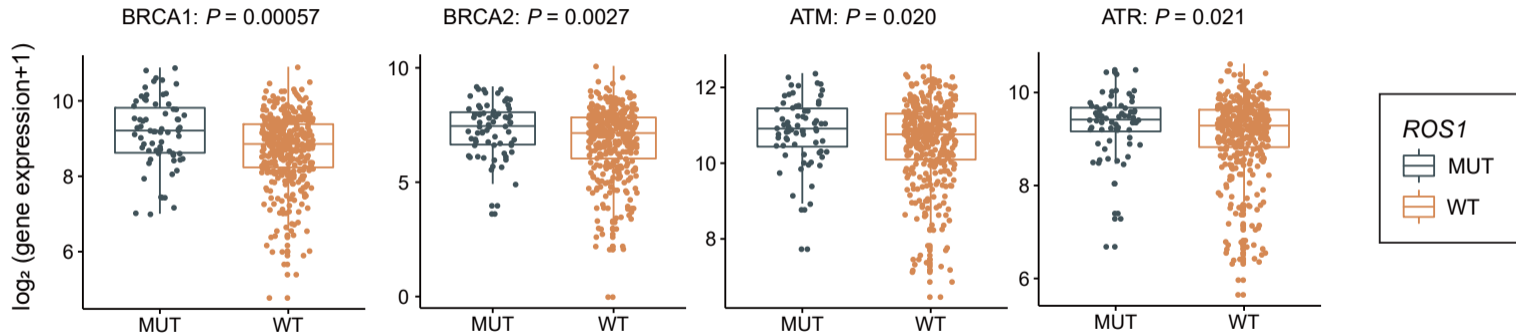

Supplement: Supplementary Figure 4 — Gene expression levels of representative genes within DNA damage repair-related and damage sensor protein-centered pathways in The Cancer Genome Atlas (TCGA) melanoma dataset according to the mutation status of ROS1. [file DataSheet_4.pdf]

**A****ROS1-MUT vs ROS1-WT**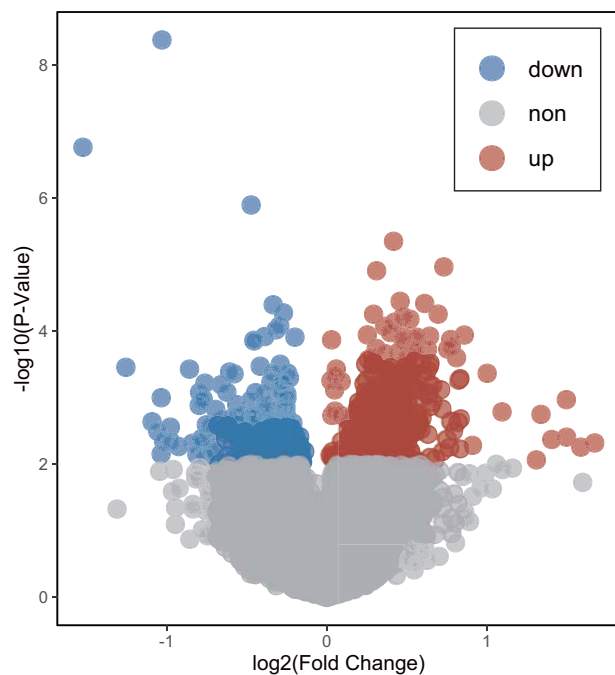**B**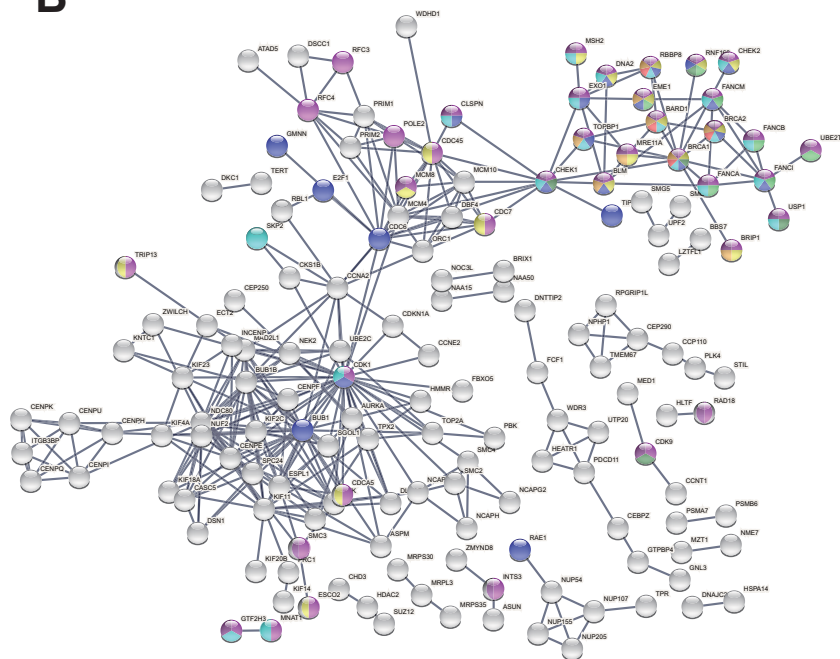**C**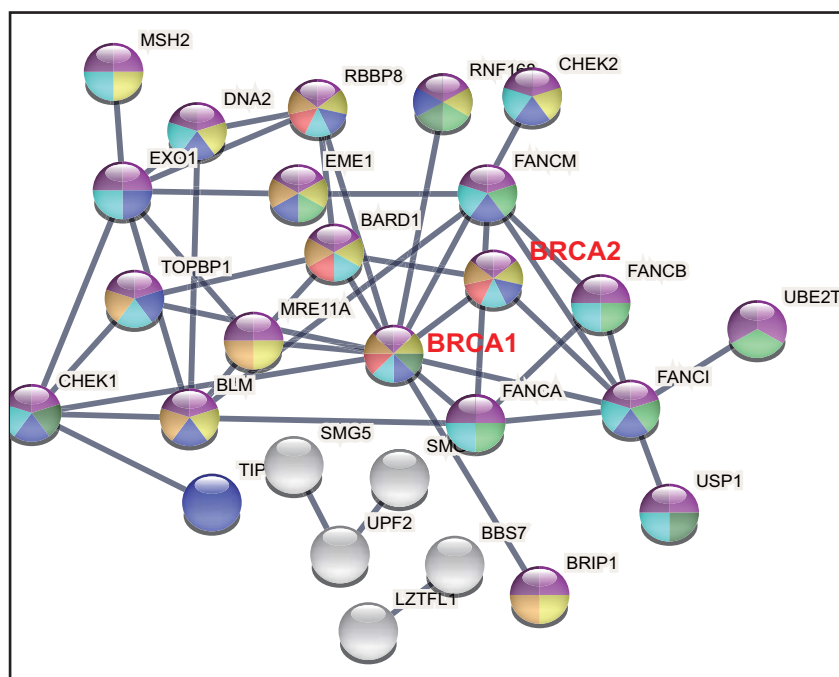

Supplement: Supplementary Figure 5 — Network analysis for ROS1 downstream mechanisms in TCGA melanoma dataset. (A) Volcano plot showing differential analysis of ROS1 mutation versus wild-type; those genes with P<0.01 were retained for network establishment. (B) Interaction network with a minimum required interaction score of 0.99. (C) BRCA-centered subnetwork. DNA repair-related molecules were color coded within the network. MUT, mutation; WT, wild-type; TCGA, The Cancer Genome Atlas. [file DataSheet_5.pdf]

**A**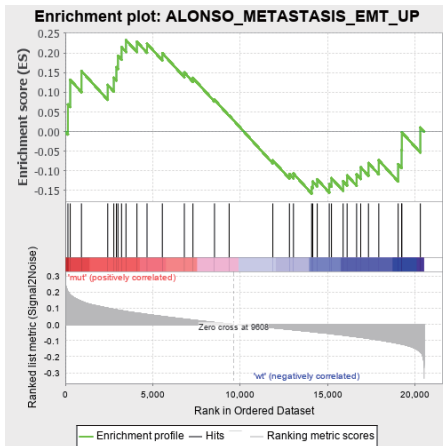

NES = 0.7815  
FDR q-value = 0.8275

**B**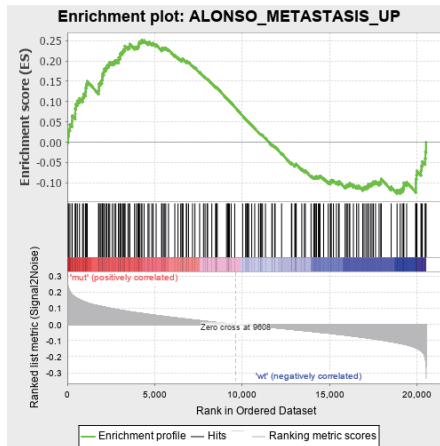

NES = 1.0692  
FDR q-value = 0.4983

**C**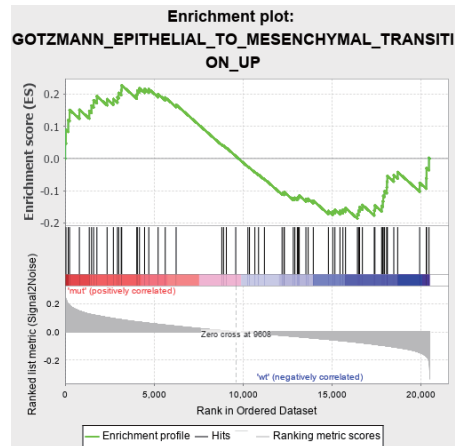

NES = 0.8038  
FDR q-value = 0.6852

Supplement: Supplementary Figure 6 — Enrichment plots of ROS1 mutation versus wild-type in biological processes concerning metastasis or epithelial-mesenchymal transition (EMT). NES, normalized enrichment score; FDR, false discovery rate. [file DataSheet_6.pdf]
